# American Dental Association

**Published:** 1895-10

**Authors:** 


### Reports of Society Meetings.

### AMERICAN DENTAL ASSOCIATION.

*August 6, 1895.—First Day.—Morning Session.*

The meeting was called to order by the President, Dr. J. Y. Crawford, of Nashville, Tennessee, at 11 o'clock A.M.

The exercises were opened by prayer offered by Dr. Hoff, and the President then introduced Dr. Sanger, of New Jersey, who made the following remarks:

Mr. President, Ladies and Gentlemen, Members of the American Dental Association,—In the name of the New Jersey State Dental Association I salute you and bid you thrice welcome to Asbury Park. When the dental student enters upon his career he has but one ambition,—that of attaining to a diploma, and with his fellows he strives through anxious years until the Dean finally notifies him of his acceptance, and his heart is filled with joy. Mr. Chairman, the New Jersey State Dental Society has this year attained to a diploma, the diploma of twenty-five years of usefulness, and a history of which it is proud. To-day, as we look you in the face, we feel we have attained the scholarship prize,—that, representing as you do the intellect and the highest intelligence in dentistry, and honoring us with the privilege of being your hosts, you have said to us, "Well done!" and you have given us the prize. We feel a pride in it, because we believe we have attained a reputation as leaders among those who have sought to make the profession what it is to-day; so we are additionally glad and peculiarly joyful as we find this sanction to our work in your acceptance of the invitation to be our guests. The latch-string is on the outside to you, and the greatest reward you can give us is to have your hand often on it, feeling assured that we will gladly greet you and give you what you wish. Again we bid you thrice welcome, with the hope that while the members of this Association are getting new ideas and tools for their professional work, the air of Asbury Park may bring new lustre to the eye, new quickening to the blood, so that in the future you may look back upon this as a beautiful oasis in the desert,—one to which your heart shall turn with delight again and again.

*The President.*—We have with us to-day a representative of American dentistry in a foreign land, Dr. Bryan, of Basle, Switzerland, ex-president of the American contingent of dentists in Europe, and we should be pleased to hear from him.

*Dr. Bryan.*—I came here expecting to be an observer, having nothing to do, and to be called on to-day for a speech is entirely unexpected. Our association in Europe has great pleasure in looking up to this society as to its father. It is a much older association than ours. The Society of American Dentists in Europe was formed about twenty years ago, as near as I can remember, on the Rigi Mountain, by Americans residing in Switzerland. They gathered there on the Fourth of July to have a little celebration, and organized the American Dental Society of Europe. It rapidly grew in numbers, and drew to its ranks members from all over Europe, and all the nations have one or more of its representatives in their principal cities. We meet annually, as you do here, and our Society is now in session at Boulogne-sur-Mer, in France. They charge me to extend to you their greetings, and hope that the members when they wander abroad will arrange to meet with them. We, as exiled Americans residing abroad, are always glad to extend the hand of good fellowship to you, and nothing would please us more than to greet you all. I thank you for the honor conferred upon me, and am glad to be with you to-day.

The President introduced Professor Dunbar, of the Dental Department of the University of California, who spoke as follows:

I represent only a small part of the dental profession in this country, and we are united by only a very slender bond. The American Dental Association has never gone farther west than the State of Missouri, and we feel that we have been slighted to some extent. We are not many in numbers, but we have an active working profession in California. I have hoped in years past that we would have this Association with us. We hope some time in the future you will come over and help us. We need encouragement. We think this sort of assistance is what the American Dental Association can furnish. I thank you for the distinguished honor of speaking for my State and city.

Dr. Brophy moved that the American Dental Association send greeting to the American Dental Society of Europe, now in session at Boulogne-sur-Mer, France. The motion was carried.

On motion, the roll-call was omitted, and the reading of the minutes of the last session dispensed with for the present.

Dr. Crouse, on behalf of the Executive Committee, reported that the working programme of the meeting would be the printed programme which was mailed to the members and distributed, except that for the purpose of accommodating Sections IV. and VII., who wished to illustrate by lantern slides some of their work, Thursday evening would be set aside for those sections. Dr. Crouse also reported about the arrangements he had made with the railroad companies as to the reduction in fare.

The report of the Publication Committee was then read by Dr. Cushing, and was followed by the report of the Corresponding Secretary, Dr. Emma Eames Chase. Dr. Chase also read a letter from the Maryland State Society asking the aid of the American Dental Association and the Southern Dental Association in establishing a dental exhibit in the Army Medical Museum and Library Building in Washington.

Dr. Morgan, the Treasurer, presented the following report:

RECEIPTS.

| | | |
|---|---|---|
| On hand last year | $1159.42 | |
| Collection at Old Point Comfort | 390.00 | |
| Dues collected since | 195.00 | |
| | | $1744.42 |

EXPENDITURES.

| | | |
|---|---|---|
| Salary of the Secretary for 1893 to 1894 | $200.00 | |
| Salary of the Treasurer | 100.00 | |
| Reporter | 125.00 | |
| Chairman of Section I. | 9.95 | |
| Chairman of Section II. | 5.15 | |
| Chairman of Section VI. | 4.00 | |
| Executive Committee expenses | 33 00 | |
| Incidental to the Publishing Committee | 1.15 | |
| Expenditures for expressage, postage, and stationery | 10.75 | |
| Columbian Dental Congress | 500.00 | |
| S. S. White Company, postage, etc., for Transactions of 1894 | 88.49 | |
| George H. Cushing, for Publishing Committee | 60.00 | |
| | | 1137.49 |
| Leaving balance on hand August 6, 1895 | | $606.93 |

Dr. Cushing announced that the following gentlemen had offered their resignations: A. W. Candless, of Chicago; J. W. Palmer, of Pittsburg; and Dwight Smith, of New York.

A motion was made to accept the same, if the gentlemen were clear on the books.

Dr. Cushing read a communication from Dr. H. E. Beach, President of the Southern Dental Society, extending his fraternal greeting, regretting his inability to be present, and inviting all the members of the American Dental Association to meet with them the first week of November, in Atlanta.

President Crawford then read his address, an abstract of which follows:

Gentlemen of the American Dental Association,—While I am not unmindful of the compliment you paid me at your last meeting, in electing me to the presidency of this honorable body, I fully appreciate the fact that the great responsibilities fully equal, if they do not exceed, the honor conferred; therefore I hope you will permit me to say that what little I have been able to do towards this grand consummation has been with a view to advance the interests of the American Dental Association.

At the last meeting some work was laid out by the appointment of suitable committees that will come up for consideration at this time. The committee to have charge of the Horace Wells Memorial Meeting at Philadelphia inaugurated the work by holding a memorial meeting in the city of Philadelphia on the 11th of December, 1894, at which time it was determined by those in attendance that it was the duty of the dental profession to erect a suitable memorial to the memory of Dr. Horace Wells as the discoverer of anæsthesia.

The notable features of that meeting were the two addresses delivered by Dr. Thomas Fillebrown, of Boston, upon the history of anæsthesia and its discovery by Horace Wells, and by Dr. James E. Garretson upon the advantages resulting from the discovery to surgical science. I recommend that the two public addresses, together with a synopsis of what was said at the banquet held in the Union League Building, be incorporated into our annual proceedings and printed as a part of this year's work of this Association.

A committee to act as a joint committee with a like committee from the Southern Dental Association was appointed to investigate the feasibility of bringing together the working material of the two Associations in order to have one thoroughly national body. As the Southern Dental Association has not held a meeting since our adjournment at Old Point Comfort, I recommend that this committee be continued until our next annual meeting, in order that the Southern Dental Association may have an opportunity, if it so desire, to appoint a like committee.

The old question of representation of dental surgery in the medical corps of the army and navy demands an earnest and united effort on the part of our profession, and especially by the members of this Association. As the most important specialty in medicine and surgery, we should demand this recognition by the governmental authorities, first, because it is a necessity to the comfort and well-being of those who bear arms in defence of our homes and institutions; and, secondly, because of the fact that it is right in every particular, and would be a means of ultimately improving the physical condition of the soldiery of our country,—a question of paramount importance in the make-up of an army of defence.

I would suggest that the strongest and best committee be created to investigate the propriety of some inquiry being made in regard to the condition of the mouth and teeth of an individual before obtaining life insurance, as the matter of proper medical examination is of supreme importance in considering the integrity and perpetuity of the life insurance companies of our country. Knowing as we do how destructive diseases of the mouth and teeth are to human comfort, happiness, and health, it is passing strange that no attention has been paid to the subject in conducting the medical examination of an applicant for life insurance.

Some of our State and local societies are attempting to have more attention paid to dental prophylactics in regard to the scholastic part of our population. I recommend such action by this Association as will further the work in this line. Particularly would I insist that we have some discussion from a dento-mental stand-point as to the propriety of regulating the time when a child should enter our public schools for instruction at the expense of the public treasury. Our system of education is sacrificing the teeth and health of our people by putting the child at school at too tender an age. A child should not be put to hard study while he is undergoing the process of tooth-shedding and tooth-eruption. The prematurely created nervous strain upon the child by overtaxing the mind is much more destructive of proper nutrition and physical development than has yet been conceived of.

As another means of instruction and advancement in the practical application of dental science to the masses of the people, I respectfully request that this Association, by resolution or otherwise, ask the medical colleges of this country to create or institute special chairs upon the subject of dental and oral surgery,—not nominal, as many schools now have, but *bona fide* chairs, so that those who expect to graduate in medicine be required to know something of the diseases of the mouth and teeth, and the deleterious influences they have in the impairment of the general health. The objects of the labors of this Association should be to encourage advancement along the lines that promise the most good to the greatest number. The nation's health and longevity is the question at issue, not the making of money by a favored few. The practical application of dental surgery and dental science is a national necessity. Many other suggestions could be made with propriety at this time for your careful consideration, but enough for the present.

I will now call your attention to the subject of my address proper,—ethics. No one question demands more honest, faithful attention at this time than ethics. In fact, the disregard of proper ethical conduct upon the part of men in all lines of business and professions is a serious menace to our welfare as a people. The only difference between commerical ethics and professional ethics is a supposed higher mental attainment of the one class over the other, and the interest involved. Hence the idea of professional men being, in addition to their usual duties, teachers in the highest and best sense of the most exalted system of ethical conduct, is apparent to the casual observer.

The correction of the many abuses that exist by reason of the disregard of proper ethical conduct cannot be accomplished at once. It must be the result of long and persistent effort on the part of such societies as this, and others of like character in other professions. Some have suggested the invocation of legal enactment by the various States in order to put a stop to the nefarious practice by many of our profession in disregarding the plain teachings of our written code. Such enactments cannot be had until we have a better public sentiment upon the subject, which better sentiment must and can only be obtained by utilizing all the forces along the line of proper education. To make myself clear upon this point, let me state that the pernicious influence resulting from the almost universal habit of the secular press of our country in advertising to the world the innumerable number of patent medicines, without any knowledge of their efficiency, is one of the most glaring and baneful violations of ethical law now in existence. It is not uncommon to see a written testimonial of prominent officials, presidents and professors of universities, giving public endorsement to valueless and even hurtful patent humbugs as medicinal agents for the relief of disease. Ethics in its highest sense teaches the duties of man to himself, to his fellow-man, to his country, and to his God. Every infraction of ethical law is bound to result in injury to some one. Taking this as the proper standard of human action, it is an easy matter to determine right from wrong in the affairs of life. A disregard of the teachings of æsthetics tends to blunt the moral sensibilities, and degrades an individual to such an extent that he is no longer suitable for a high and honorable profession. If the dental surgeons of the United States would unite in demanding the enactment of such laws as would require all men proposing to engage in the practice of dental surgery to observe proper ethical conduct, it would be but a short time before the statute-books of all the States would be graced with laws that would annihilate the quack and the mountebank. As dental surgery is one of the most æsthetic professions, it should be the most ethical in its conduct, thus giving the world a noble example of what it is to do right in all things. As an immediate remedy for the future, I would suggest that all of the reputable schools be requested to formulate a uniform oath or obligation, to which each student shall subscribe before a proper State officer before being permitted to enter a dental college or receive the degree of doctor of dental surgery. Every violator would be subject to indictment for perjury by the grand juries of our States, which would deter many from wrong-doing. I would further suggest that this be required of the schools before being eligible to membership by representation in this body.

It is my painful duty to call your attention to the fact that some of those who were with us at our last meeting, as members of this Association, have gone to "the undiscovered country from whose bourne no traveller returns." Upon the list are the names of those who have been prominent workers in the interests of the American Dental Association. I recommend that a suitable memorial be prepared and placed upon our record-book in honor of their memory.

Dr. Walker moved that the President's address be referred to a committee to report at a later session.

Drs. Walker, of New York, Crenshaw, of Atlanta, and Fuller, of St. Louis, were appointed as such committee.

Dr. Crouse stated that the afternoons of each day had been set aside for the work of the sections, and the chairman of the various sections announced the place of meeting of the same.

Dr. Cushing moved that all visiting dentists not members of the Association, and all physicians present, be invited to the privileges of the floor. Such motion was carried.

Dr. Ottofy, on behalf of the Committee on Credentials, read the names of the delegates who had presented credentials from their societies.

A communication was read from Mr. Ten Broeck, the mayor of Asbury Park, who regretted his inability to be present, but sent his greetings to the American Dental Association, and presented the freedom of the borough. The same was received with thanks.

*Dr. Rhein.*—I wish to know if any arrangements have been made for giving clinics before this Association. I would like the information to be given to the Association at this session, because I have been informed by a number of gentlemen that they have been invited to give clinics, and at our last meeting we voted that we would not have any clinics.

*Dr. Crouse.*—There will be no clinics given before this Association, except under section work. The vote of last year will be obeyed.

*Dr. Fillebrown.*—I would like to offer the following resolution:

*Resolved*, That this Association believes the conferring of honorary degrees in dentistry is detrimental to the interests of the profession of dentistry, and this Association hereby expresses its disapprobation of the practice.

Dr. Walker moved that the resolution be referred to a committee of three, to be appointed by the chair.

*Dr. Bogue.*—I offer the following amendment to that motion: That that committee consist of dentists who are regularly graduated in course from some dental college, and who do not belong to any college faculty.

*Dr. Barrett.*—I cannot conceive the propriety of that. There are reputable men in the profession who are not graduates, and who have a perfect right to vote on any question which affects the interest of the profession. No question which so deeply affects the whole profession should be sprung upon a body like this without due consideration.

Upon motion of Dr. Stainton, Dr. Walker's motion and the amendment were laid on the table.

Dr. Fillebrown's resolution was then carried.

Adjournment to 7.30 o'clock this evening.

*August 6, 1895.—First Day.—Evening Session.*

The vice-president, Dr. Watkins, in the absence of Dr. Crawford, who was ill, called the meeting to order.

The secretary read the minutes of the morning session, which after slight correction were approved.

Dr. Crouse moved that the Committee on the Report of the Horace Wells Memorial and the Committee on Census be made a special feature for Wednesday afternoon at three o'clock. Motion carried.

The secretary moved that Dr. Harlan's paper, entitled "Antiseptic Surgery," go over until next year, to be read then as the annual address; but Dr. Peirce suggested that it be read by title and referred to the Publication Committee, to be printed in this year's Transactions, and the amendment was accepted.

Dr. Ottofy, in his report of Section II., stated that forty-eight dental colleges are now in active operation and granting degrees. The transactions of the World's Columbian Dental Congress, in two volumes, appeared last December, and are without doubt most important contributions to dental literature, and no library is complete without a copy of the work. There has been an attempt made to introduce the name "stomatologist" instead of "dentist," and one journal has changed its name accordingly. So far as known to the writer, none of the dental journals have suspended publication during the year. There has appeared a journal called

*Dental Digest*, which intends to present each month a *résumé* of all that appears in the dental journals of the world, thoroughly sifted, and the most essential and important parts presented to the readers of the *Digest*. If this can be done, it will certainly be a great addition to our dental literature.

The report was received.

Dr. C. W. Stainton, of Buffalo, then read a paper entitled "Ought the Formation of Dental Schools to be limited?" which was illustrated by charts giving statistics as to the number of physicians and dentists in relation to the entire population. An abstract of the paper follows:

Any one who has thoughtfully listened to or read the annual reports of the chairman of this section (Dental Education) for some years past must have observed that regret has been expressed that dental educational institutions are being multiplied so rapidly. Conversation with our most experienced educators shows almost universal concurrence in this view. Are we overdoing the formation of dental schools and the production of dentists? The matter is worthy of serious consideration and study, in order that proper checks and remedies may be used in time to correct this evil, if it be an evil, before it becomes too late. In casting about for some starting-point, some foundation which all will agree upon as fixed and sure, two premises will be acknowledged by all as conceded.

First, that in all civilized countries every individual in the community is a part of the medical constituency. In this the volition of the individual does not govern, but the public welfare will compel medical treatment when the public health is in any way threatened. Every individual, no matter what the color, age, sex, or condition, comes sooner or later under the care of a physician, and constitutes a part of the medical constituency.

Our second premise is that in medicine, which is as old as the human race, the law of supply and demand has long been practically established. There may be more medical men seeking practice than can find it. This profession, like the law and the ministry, is so overcrowded that the survival of the fittest is being constantly illustrated. The number of medical men in any community or country is a fair measurement of the number needed in that community or country.

I have taken a great deal of pains to prepare a table of fifty cities in the United States, including of course all the principal ones, giving the population, number of physicians and dentists, and the average number of inhabitants to every physician and dentist. This table has been obtained in several ways, largely by correspondence with dentists in these cities. An attempt has been made to procure from many dentists estimates of the percentage of the population in their city who employ dentists, but the attempt has not been very successful. Nobody knows positively anything about it, and many have never given it a thought. The dental ranks are as full to-day in the United States, in proportion to the demand, as the medical ranks. The overcrowding of our ranks in any locality is prolific of cheap practitioners, and the lowering of the standard and character of our specialty.

But can we forbid to any young man the study and practice of dentistry? Certainly not. The raising of the standard of admission to our dental colleges, and the lengthening of the course of study so that it takes as long and costs as much to enter dentistry as medicine, will do much to deter from the hasty entering of dental schools. The multiplication of dental schools is the chief danger in this direction. Half of the medical schools organized in this country have died out, and the experience of our medical brethren ought to be a lesson to us. Dental schools are not being formed now from any need felt for them as an educational necessity. Two impulses now control this matter,—impulses unknown in the beginning,—

First, personal ambition to have a position in and be connected with a dental school for the prominence it is supposed to give; secondly, a purely commercial spirit on the part of medical schools to have a dental department.

The National Association of Dental Faculties ought at once to serve notice "that hereafter no dental school will be accepted, under any circumstances, unless the consent of the Association has been first asked and received for its formation." Would it be too great an assumption of power for the National Association of Dental Faculties to do this? Certainly not. The Faculties Association has proved its character and wisdom beyond the largest expectation. A body which can modify and improve at every point its own interests, constantly bettering the condition of everything it touches with its sceptre of power; that can chastise its own members, no matter how old and intrenched in position,—yea, better, can reach outside its own circle, and correct and punish the unseemly behavior of older institutions who wish to lord it over us; an institution which has done all this without even the smell of fire being found upon its garments need not hesitate to undertake any reform that is needed.

Dr. Louis Jack, of Philadelphia, then read his paper entitled "Should not the Increase of Dental Schools be restricted?" of which the following is an abstract:

There has been a feeling on the part of both the National Association of Dental Examiners and the Association of Faculties that the increase of schools was proceeding at too rapid a pace, and the sentiment was expressed that a greater requirement than additional schools would be an extension of the period of instruction and an enlargement of the curriculum of the colleges already established. A very important condition appearing in conjunction with these sentiments is that the newer schools and those under organization are not being installed with the equipment, the facilities for instruction, the fulness of faculties, and such other instructors as they should have, some of these schools being such in name only, rather than in fact. It may be of interest to explain the value of the diplomas of the unorganized schools. While they have legal value in the States where they are, they give no *prima facie* right to practise anywhere else without examination by the State Boards, and in some States they would not have the privilege of being examined. A manifest tendency of the present period is in the direction of the establishment of dental departments in connection with medical schools. I have no disposition to inquire closely into the motive for this association of dental with medical schools, but we have to deal with a new and dangerous situation. It is palpable that the correct motive underlying all attempts at teaching should be the provision for the fullest and the most varied instruction pertaining to the subject to be taught. In reference to dentistry this now requires an installation far beyond what the ordinary medical schools can furnish.

If the standard in the acceptance of new schools be made as high or higher than has been reached by the most approved among the older ones, the process of levelling upward will then take place, instead of a general lowering of the standing. The dental degrees of America, as is painfully apparent, are being discredited in Europe, for the reason that the preliminary requirements of the students and the curriculum are not sufficiently advanced. This sentiment should be corrected by conforming to the standards which will do this. In case some brake is not put upon the addition of dental schools which are not of high order, the prejudice against the dental institutions of this country must further increase. It must be considered unfortunate that it is a difficult matter in this country to control the establishment or the conduct of educational institutions. The States have no principle which can be exercised to regulate the quantity of schools. In Europe these questions are carefully governed by the state, which provides laws, grants privileges, and makes rules regulating such public affairs. Here the crystallization of sentiment into law is more lax, and therefore we have to depend upon the development of opinion to assist and support such bodies as the Association of Faculties and the National Association of Dental Examiners in the formation of such rules as shall guard the interest of all concerned, and to aid in checking the degradation of our profession.

*Dr. Ottofy.*—Inasmuch as Dr. Jack's paper is a reply to Dr. Stainton's paper, I would move that we now hear Dr. Barrett.

Motion carried.

Dr. Barrett, of Buffalo, then read a paper entitled "Whither are we drifting," an abstract of which follows:

Most dentists accept as an axiomatic fact the assertion that dentistry is an integral part of medicine when properly practised, and most physicians promptly admit it. There is year by year a constant extension of the several fields or specialties into which medicine is divided, and consequently the future demand for dentists will increase at a greater ratio than will the population. We now have nearly fifty colleges, each of which is doing something towards extending the field of dentistry. Each will widen its curriculum and broaden its teachings through the natural emulation that must and shall exist. Dentists this year are performing operations and treating pathological conditions unthought of last year. The dentist writes his prescription for and directs the treatment of a large and constantly augmenting list of human ailments. His field has increased a hundred-fold. It is true that dentists have greatly multiplied in number, and we constantly hear from some of the old-time practitioners who have not broadened their practice with the advance of modern ideas that the colleges are turning out graduates at a rate that must soon make their number greater than that of their patients. The profession is growing in importance, and we can easily see the time when there will be fifty thousand dentists in this country. What are we doing towards securing an organization that shall make of them the most effective body of practitioners that the world knows?

The mere desire for the listening to and the debating of scientific papers will never be sufficient to call together any great proportion of our numbers. There must be some other attraction. A considerable portion of those who are at Asbury Park to-day come that they may renew old acquaintances and enjoy old friendships. It is the hundred or so of these old members who give the main interest to our annual gatherings. When we meet at some extremity of our great country, as at Minneapolis, and draw in a throng of new members, but a few of them continue with us, because the next year they would be compelled to travel a long distance, only to find themselves among strangers. It is then evident that something must be done to promote the communistic side of our meetings. It seems to me this must be done by division rather than by wider aggregation. We should have a number of great societies, meeting annually, with delegates from each of these to form one central association, which may meet at either a fixed point or itinerate among the different sections,—one for the East, one for the West and Northwest, one for the South and Southwest, and one for the transmontane portions of our country. But the American Dental Association has not yet served its full purpose, and I do not propose to abandon it. What can we do to make it more effective?

I hope that the matter will be thoroughly considered this year, and that we shall at least take some steps to improve the social condition; that we shall strive to get better acquainted, and bring less of personal interests to be served; that we endeavor to confine the mercantile spirit to the manufacturers and dealers in dental goods, and discourage it among ourselves; that we shall earnestly and honestly strive to approach the consideration of these questions in a broad, catholic spirit, with due charity for the weaknesses of others, and with the feeling that because our brother does not view matters from our own particular stand-point he is not therefore necessarily an insincere and dishonest rogue.

DISCUSSION.

*Dr. Fillebrown* (of Massachusetts).—I want to say a few words about Dr. Stainton's paper. The impression is generally extant, I think, that it is within the power of the National Association of Dental Faculties to forbid any more dental schools. We do not have to pay the bills, and we cannot forbid it. A school that came before the Association at this session for admission said, "We have our equipment and we have money enough behind us to make it all we want, and we shall do it." We have no power over them, of course. Look at it from the light of actual fact, and you will give us a little more sympathy. You will not make such great demands. In regard to the number of medical practitioners, I think the large army of medical men are not considered in this report; consequently there are a great many more physicians to the patient than this table represents. There are a great many more dentists needed. I have not had time to figure out the increase from year to year, but I think it will prove larger than the increase of graduates. The graduates from year to year are not enough to supply the increase; consequently we are not yet overstocked. The propriety of dental examiners examining graduates has been questioned, as though that were a great hardship; that because a man graduates in one State he cannot go into another State without being re-examined. To my mind that is no objection. The State of Massachusetts has a law for lawyers and for dentists, and I think it applies to medicine, that every graduate must submit to an examination by the Examining Board before he can have a license to practise in the State of Massachusetts. Every man who graduates from the law-school of Harvard University must go before the officials and pass an examination. It is a good whip to hold over the schools. I hope it may not be rescinded, and I wish it might obtain in every State of the Union.

*Dr. McManus* (of Connecticut).—I have been specially interested in the papers this evening, for the reason that I have been engaged in a legislative fight this season. In the State of Connecticut, at the meeting of the last Legislature, a bill passed the lower House to incorporate the Connecticut College of Dentistry. The Board of Dental Commissioners knew nothing about it, and I do not think twenty dentists out of three hundred and seventy-five in the State knew such a thing was to be asked for, or thought such a college was needed in the State. After seeing the announcement in the paper that the bill to incorporate this college had passed the House, the recorder of the Dental Commission and myself went to the State House to see the governor, and talk with him regarding the incorporation of this college. The governor suggested that the bill be returned to the committee and that the dentists should have a hearing. In the mean time our State Society had its annual meeting, and we appointed a committee to appear before the Legislature and ask for a hearing. The Society appointed a committee of three to appear before the Legislature, and that committee invited three of the ex-presidents and some other gentlemen to appear at the meeting. I was asked to present the protest. My protest took exactly eight minutes to read. The same committee reported again in favor of the establishing of this college, and the lower House passed it again. Then we had to go to the Senate. Fortunately on the staff of the governor of our State, Dr. Graham is the adjutant-general, and they listened to him. Through his influence and that of others we managed to prevent the incorporation of that school. We did not want an institution incorporated that in the nature of things could not be a success.

*Dr. Stockton* (of New Jersey).—Professor Buckingham, whom we all loved and honored, said once to this Association, " We do the best with the material that has been sent us that is possible to do." That may have been true in his day; I doubt very much if it is true to-day. The only point I wish to raise to-night is this: Cannot the colleges of this country turn out gentlemen? If they did, the snub put upon my friend Dr. McManus would never have been uttered. Can there not be some restriction thrown out that a graduate will not put up a sign like this, " Hanks yanks teeth?" That is only one instance, and you know it occurs all over the land that men advertise the best set of teeth that can be furnished for five dollars, warranted to last a lifetime. I claim that the colleges can very largely remedy this evil by at once saying, when the fact is brought to their knowledge, that a man who does this sort of thing shall at once have his diploma withdrawn. He may own the diploma, but they can put a brand upon him that will make it very unpleasant for any man to assume that position. He gets a diploma and hangs it upon the wall, and claims that he is a good dentist and that a good college has qualified him to practise dentistry, and is privileged to do as he pleases. If the dental colleges would put a brand upon such men, all such practices would soon cease.

*Dr. Rhein* (of New York).—While listening to the paper of Dr. Stainton, of Buffalo, and some of the discussion that has taken place on these papers, it appeared to me as if I were at a meeting of a plumbers' or a central labor association. It does seem that it is a spirit of illiberality that would make us attempt to restrict the entrance of men into our calling. On the contrary, as members of this Association we cannot fail to recognize the enormous amount of poor timber that there is among men who call themselves dentists, and who swell this list of names that is brought before us as practising upon the mouths of patients throughout the United States. How are we going to elevate the character of the profession by putting up a Chinese wall about us and endeavoring to keep out practitioners, as has been done by a great many States in this country by the laws which have been enacted, some of which will not bear judicial inquiry. To come down to the basis of the figures that present themselves here before us, I challenge the point that they meet the question of supply and demand. The tendency of this country is to increase the general education of the people, and as it increases so will they learn the value of taking care of the dental organs. As it is, the present number of dental schools, with the number of matriculates they have, is totally inadequate to supply the proper number of dentists demanded in the United States at the present time. It is one of the worst blots that can be put upon this Association to go out, either in the public press or before the civilized races, as taking the position that some of these papers do. The question as it presents itself to me is not that there are too many good dentists, but that there are too many poor dentists. That is the question we have to combat. Can we make better dentists out of men who have become wedded to their methods of practice? It is of no avail to prescribe the oath that was formerly administered to every man who received his diploma. That will not aid us among the large mass of men who have their *clientele* and go on year after year destroying the teeth that come into their offices. What will improve dentistry will be the establishing of better dental institutions than we have to-day. It is not our place to find fault with any number of men who wish to found a college and connect it with some university or some permanent institution, and wish to put it upon a plane beyond that of those who are competitors in the field of learning. That spirit should be applauded by this Association, for it is the only way in which better men can be turned out, the only stimulus that will make the other institutions come up to a higher standard of dental requirements.

*Dr. Flagg* (of Pennsylvania).—The subject, it seems to me, is one of the very greatest moment to our profession. The entire time of this organization could probably not be better spent than by having a full, free ventilation of opinion in regard to this matter. I have not a doubt that the various papers that have been read on this subject this evening have struck upon the mind of those present each in a different way, but all tending towards one direct end. It perhaps would be better not to say one word in regard to this matter, for I am so unfortunately constituted that I almost always stir up contention. I seem to be a sort of firebrand, and therefore it is my disposition to remain quiet until I am not able to do so any longer. As I view it, a house divided against itself cannot stand. I have endeavored to urge that dentistry with me is first, greatest, grandest, best all the time. Dr. Jack says there is a great danger threatening our profession. What is that? Are we a profession? Are we united in regarding ourselves as a profession? A very few minutes after that he spoke about the ability on the part of certain universities, because of their peculiarities of education and their great facilities in regard to educating our "specialty." It seems to me that there is the rock on which we split. We are all striving truly for the good of our profession. We want a more generally educated profession. We want to recognize that they are gentlemen who are turned out from the colleges. I think a university would be the very thing to educate men for the profession of dentistry, but not through the agency of their medical chemists and the other men.

I do not like to speak of the commercial aspect, because if there ever was a man who went into the profession with a twofold idea, it was myself. I wanted to do what I could for education; I wanted to do what I could in regard to development, and I wanted to pay for it. I want to be paid for what I do, too. There are scores of men like myself who want big classes and good pay, and they want their graduates to be creditable all through the United States and to be able to go before any boards. I rely upon the boards. I took that ground in our Faculty, and when opposition was made that it was rather discreditable that our diplomas should be looked at sidewise by State boards, I said, "It is the presence of State boards that will prevent them from being looked at sidewise." I think State boards are the foundation for the progress of dentistry, and for a man to be examined from State to State appears to be the proper thing to do at the present time. There are many questions in this matter, but they are all centred upon this one thing: that if the medical staffs and the medical colleges still hang on to the idea that this profession of dentistry is going to be elevated as the wiggle-waggle tail of medicine, we will not make any progress.

Dentistry is so great that no one of us is a thoroughly well-educated dentist. I challenge any one to get up and say he knows everything about prosthetic dentistry, from vulcanite work to continuous gum; that he knows every medicine known to dentistry and all about them, and that he knows everything about dentistry from A to Z. If he is here, let him get up, so we can look at him.

*Dr. Lenox* (of Toronto, Canada).—The difficulty, to my mind, is more with the people than with the schools. We have for years and years talked about educating the people, but it is a very difficult matter to educate the people. I believe in thorough education of the dentist, and too much cannot be done in that direction. It has been said that men will be graduated from institutions of good standing, and they will advertise to do thus and so, and the people patronize them. Educated dentists will take their stand among educated people, but we find that many we are called upon to treat are not intelligent people. The consequence is that when a man advertises to do a certain thing by the use of a certain article,—to extract or fill teeth without pain,—nine people out of ten will go to that man. Can the people be educated? We have been trying to do it for many years, but it seems to be a difficult matter.

*Dr. Bogue* (of New York).—I find myself agreeing mainly with what Dr. Flagg has just said. I am asking the question why are these papers read before us at this session this evening. It seems to me that they are read because the Association possesses moral weight. I was deeply impressed by Dr. Stainton's figures, as I have been deeply impressed by another fact,—namely, that I know quite a number of persons who are utterly unable to speak the English language with propriety, and yet this year have been made doctors of dental surgery by our colleges. I presume every one in this room is familiar with one or more of such cases. It is in vain for us to apply to the colleges to recall their diplomas after they have been given out, unless a sufficient moral force is brought to bear upon those colleges to do that. This Association is certainly able to exercise an influence of which it does not seem to be aware. Not many years ago, through a resolution, it called into being the Association of Dental Faculties.[1] At the present moment there is in existence an association which, it seems to me, can be slightly directed by this Association, which can have a very great influence upon the questions that have been brought before us, and that is

---

[1] This is a serious error and requires correction. The National Association of Dental Faculties *was not* "*called into being*" by the American Dental Association. We are not aware that a resolution was ever passed by this body requesting the formation of such a controlling association, and had it been there was no central power qualified to receive it. The colleges of the period were isolated and very independent, recognizing no law but their own governing Faculties. The Baltimore College of Dental Surgery, under the leadership of the late lamented Dr. Winder, suggested organization to the three colleges of Philadelphia, and at a conference held in the latter city a call was issued, which resulted in the formation of the powerful body known by the above name.—[Ed.]

the National Board of Dental Examiners. Between the National Board of Dental Examiners and the Association of Dental Faculties this question can be settled, but our moral weight as a representative association from all the States of the Union will have to be brought to bear upon those two bodies. If we have a National Board of Dental Examiners with a formulated standard of dental education, then the colleges may safely and properly consign to that board the examination of their students. Then only may the States properly accept the diplomas each of the other. To-day New York has the most illiberal law that I know of. It is a law which I suppose one of the speakers alluded to when he said it had not been adjudicated, and might be upset. But while illiberal and not yet adjudicated upon, it is a law which I believe was earnestly and conscientiously formulated for the purpose of lifting the standard of dental education in that State. It is not possible for a person in New York to commence the study of dentistry until he can read and write the English language with propriety. If this body will give its moral support to such a condition as will enable the National Board of Dental Examiners to establish a status of dental education, then we shall reach something that is practical. I earnestly hope we will be able to do that.

A motion was made to close the discussion, but Dr. Shepard moved to amend that by suspending the discussion until to morrow morning.

Dr. Fillebrown was appointed in place of Dr. Walker on the committee to report on the President's Address.

The mayor of Asbury Park very kindly sent the band to the convention hall to play for the meeting.

Adjournment until nine o'clock Wednesday morning.

(To be continued.)

